# Aripiprazole modulates splenic microbiota and immune response elicited by single prolonged stress in rats

**DOI:** 10.1007/s43440-026-00882-2

**Published:** 2026-07-20

**Authors:** Peter Vargovic, Andrej Tillinger, Lubica Horvathova, Lila Dziewiczova, Jana Osacka

**Affiliations:** 1 Institute of Experimental Endocrinology, Biomedical Research Center, Slovak Academy of Sciences, Bratislava, 845 05 Slovakia; 2 Faculty of Natural Sciences, Department of Animal Physiology and Ethology, Comenius University in Bratislava, Bratislava, 842 15 Slovakia

**Keywords:** Post-traumatic stress disorder, Single prolonged stress, Aripiprazole, Spleen, Bacterial translocation, Immune response

## Abstract

**Background:**

Post-traumatic stress disorder (PTSD) is associated with dysregulation of the hypothalamic–pituitary–adrenal (HPA) axis and the sympatho-adrenomedullary system, leading to immune imbalance and alterations in microbiota composition. Stress-induced disruption of intestinal barrier integrity may promote bacterial translocation to peripheral organs, including the spleen. Aripiprazole (ARI), an atypical antipsychotic proposed for PTSD treatment, also modulates the immune system and microbiota. This study investigated the effects of ARI on splenic microbiota composition and splenic neuroendocrine and immune responses in an animal model of PTSD.

**Methods:**

Rats were exposed to the single prolonged stress (SPS) paradigm to induce a PTSD-like phenotype and treated intraperitoneally with vehicle or ARI for 28 days. Anxiety-like behavior was assessed using the elevated plus maze. Splenic microbiota and gene expression were quantified by real-time PCR in isolated splenocytes following ex vivo stimulation with lipopolysaccharide or phorbol 12-myristate 13-acetate (PMA)/ionomycin.

**Results:**

SPS and SPS + ARI reduced the abundance of the phylum *Bacteroidetes*. SPS increased *γ/δ-Proteobacteria* and *Lactobacillus* abundance, effects attenuated by ARI. The presence of specific splenic bacteria correlated with anxiety-like behavior. While lipopolysaccharide-induced responses were unaffected, splenocytes from SPS-exposed rats exhibited increased expression of Th1- and Th17-related genes after PMA/ionomycin stimulation; this effect was reversed by ARI.

**Conclusion:**

ARI modulates SPS-induced alterations in splenic microbiota and attenuates heightened Th1- and Th17-associated responses, thereby contributing to the restoration of immune balance. Our findings underscore involvement of the gut–spleen–brain axis in PTSD pathogenesis and immunosuppressive/ microbiota-modulating effects of aripiprazole on splenic immune cells.

**Graphical Abstract:**

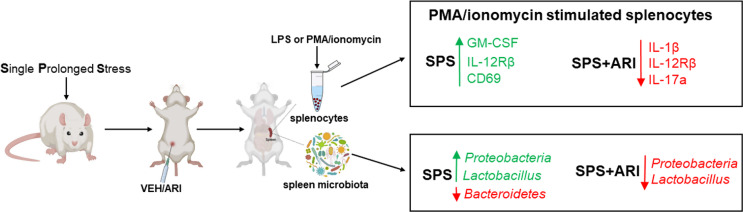

## Introduction

Post-traumatic stress disorder (PTSD) is a heterogeneous psychiatric disease that may develop following exposure to a severe traumatic event (i.e., death or threatened death, actual or threatened serious injury, actual or threatened sexual violence) and is characterized by re-experiencing the event, avoidance of trauma-related cues, numbing, or hyperarousal. Alterations in neuroendocrine, psychophysiological, and neurobiological systems play a role in the etiology and maintenance of PTSD [1]. Dysregulation of the hypothalamic-pituitary-adrenal (HPA) axis is a well-established biological feature of PTSD that results in altered cortisol/corticosterone (CORT) plasma levels, activity, and consequently in insufficient regulation of immune function and excessive inflammation [2–4]. An increasing number of studies have shown that individuals with PTSD exhibit significantly elevated blood levels of inflammatory markers, such as interleukin-1β (IL-1β), interleukin 6 (IL-6), tumor necrosis factor α (TNFα), and C-reactive protein [3].

The signaling network among the brain, spleen, and gut seems to represent an important interaction pathway impacting multiple aspects of immune functions and diseases. Changes in the spleen induced by stress are accompanied by alterations of immune cells, cytokine levels, and other molecules released into circulation and other organs in the body. Moreover, changes in the spleen’s immune cell activity appear to be linked with PTSD, too [5, 6]. The spleen is a primary source of T-lymphocytes producing interleukin 17 A (IL-17 A), which has emerged as a key player in the dysregulation of the immune system associated with PTSD. Human and animal PTSD studies point to increased plasma levels of inflammatory T helper 17 (Th17) cells, their main pro-inflammatory cytokine derivative IL-17 A, and the importance of neural and immune-derived adrenergic signaling in IL-17 A production from T-lymphocytes [7]. Moreover, it was shown that in PTSD patients, increased plasma IL-17 A levels and cluster of differentiation 4⁺ (CD4⁺) Th17 phenotypes correlate with PTSD severity [8].

Growing evidence reports gut microbiota alterations in individuals with PTSD [9–12]. Stress has been shown to cause imbalance in microbial composition, stimulate the intestinal epithelium to produce pro-inflammatory cytokines, and lead to altered permeability in the intestinal tract and excessive antigen trafficking and inflammation [13]. This “leaky gut syndrome” is associated with increased translocation of gut bacteria, which consequently reaches the liver, spleen, or other tissues. Microbial products like lipopolysaccharide (LPS) enter the bloodstream and trigger systemic inflammation, with the suggested role of the spleen in amplifying the immune responses. Thus, intestinal bacteria, individually, as consortia, as well as through their metabolites, modulate the immune response and thus contribute to the development and maintenance of PTSD symptoms [14].

Atypical antipsychotics, including aripiprazole (ARI), represent one of the possible choices in PTSD treatment [15]. Previous studies indicate that ARI may affect various immunological phenomena managed by B cells, T cells, and macrophages [16], act as a free radical scavenger, gastric protector, exert anti-inflammatory properties, and target spontaneous production and secretion of a range of cytokines and chemokines [17]. It has also been shown to exhibit anti-inflammatory effects on TNFα, interleukin 13, or IL-17 A [18]. In an ex vivo model of the human colon, ARI induced alterations in human gut microbiome composition and metabolic profile, eventually leading to gut dysbiosis [19]. The effect of ARI on gut microbiota was also observed in rats, where 4-week ARI treatment modulated the relative abundance of individual *Firmicutes* genera and increased ileal but not colonic permeability [20].

Based on the above evidence, we hypothesized that single prolonged stress (SPS) -induced PTSD would promote a pro-inflammatory state associated with dysregulation of the microbiota–immune axis. This state is expected to involve increased translocation of bacterial components to the spleen, enhanced T helper 1 (Th1) and Th17-mediated immune responses, and alterations in splenic neuroendocrine function. Furthermore, we hypothesized that ARI treatment may modulate these effects by attenuating inflammation and influencing spleen microbiota composition.

Accordingly, the aim of this study was to investigate changes in total and selected bacterial 16 S subunit of ribosomal ribonucleic acid (16 S rRNA) in the spleen using the SPS animal model of PTSD, both in the presence and absence of ARI treatment. In addition, we aimed to assess splenic neuroendocrine function and characterize immune and inflammatory responses in isolated splenocytes following ex vivo stimulation with LPS or phorbol 12-myristate 13-acetate (PMA) and ionomycin.

## Materials and methods

### Animals

In the experiment, 30 male Sprague-Dawley rats (Charles River, Germany) weighing 176–200 g upon arrival were used. The rats were housed in transparent cages with bedding, two rats per cage, in a controlled environment (22 ± 2 °C, 12 h light/dark cycle, lights on at 7 a.m.) with food and water provided *ad libitum*. Experimental procedures were performed in accordance with the Council Directive 2010/63EU of the European Parliament and the Council of 22nd September 2010 on the protection of animals used for scientific purposes and approved by the Committee of the State Veterinary and Food Administration of the Slovak Republic (Approval protocol number 5541/2023 − 220).

### Experimental design

The rats were randomly assigned to three experimental groups (*n* = 10 per group):

(1) VEH – non-stressed rats intraperitoneally (ip) injected with vehicle (VEH).

(2) SPS – SPS-exposed rats ip injected with VEH.

(3) SPS + ARI – SPS-exposed rats ip injected with ARI.

To induce PTSD-like symptoms, we used an SPS model as described before [21]. Rats were restrained for 2 h in plastic restrainers. Afterwards, they were exposed to 20 min of forced swimming in a glass cylinder filled with water (22 ± 2 °C) for two-thirds. Then the rats were dried and allowed to recuperate for 15 min. Next, they were exposed to ether vapor until loss of consciousness and finally transferred into the clean cages, 2 rats per cage. SPS protocol was followed by 14 days of sensitization period necessary for the development of PTSD-like symptoms, during which rats were undisturbed and only water and food were replenished. After 14 days of sensitization, they were injected ip once daily for 28 days with either VEH (2% Tween20 in saline, #85113, Sigma-Aldrich, Germany) or ARI (5 mg/kg dissolved in VEH, #AB120764, Abcam, USA). The ARI dose was selected based on previous literature [22]. During this period, rats were habituated daily to the presence of the experimenter and handling procedures. Consequently, the rats were subjected to the elevated plus maze (EPM) and euthanized 7 days after the last injection.

### EPM test

All rats were tested in the EPM to assess the level of anxiety-like behavior according to the previous protocol [23]. The open and closed arms of the maze were 50 cm above the floor, 50 cm long, and 10 cm wide. The rats were tested for 5 min. After each individual trial, the maze was cleaned with 60% ethanol. Rats´ movement was recorded with a digital camera, and individual sessions were analyzed with the ANY-maze Video Tracking System 7.1 (Stoelting Co., USA) computer software. We analyzed the number of entries to the open arms (OA) of the maze and the time that rats spent there.

### Isolation of splenocytes and ex vivo stimulation

The spleens were removed and cleaned of fatty tissue. After dividing into three parts, two parts were frozen in liquid nitrogen, and one part was used for the isolation of splenocytes. This portion was pre-collected into tubes with 0.5 ml RPMI cell medium (RPMI 1640 containing 25 mmol/l HEPES and L-glutamine, #BW12-115 F, Lonza, USA) with 10% fetal bovine serum (#17192345, Cytiva Hyclone, Austria). The tissue was pressed between two glass slides in a Petri dish, then homogenized by repeated pressing using a 1 ml automatic pipette and filtered through a 70 μm cell strainer (#229483, Celltreat, China). The suspension was transferred to a 1.5 ml test tube and centrifuged for 10 min at 300 × g, 4 °C. Red blood cells were removed using red blood cells lysis buffer (#420301, BioLegend, USA) and twice washed. Samples were incubated at 37 °C for 3 h in the following experimental groups:

(1) Control: added 10 µl of water.
(2) Stimulated with LPS: added 10 µl of LPS solution from *E. coli* K-235 (**#**L2143, Sigma-Aldrich, USA) to a final concentration of 10 µg/ml.
(3) Stimulated with PMA/ionomycin: Added iBioscience Cell stimulation cocktail (**#**00-4970-93, Thermo Fisher Scientific, USA) to a final concentration of 81 nmol/l PMA and 1.34 µmol/l ionomycin.

After the incubation, the samples were spun at 300 × g, the pellets frozen in liquid nitrogen, and stored at -80 °C.

### RNA isolation, reverse transcription, and quantitative real-time PCR

Total RNA was isolated using the TRI Reagent (**#**TR118, MRC Inc., USA) according to the manufacturer’s protocol. RNA concentration was determined using a NanoDrop 2000 spectrophotometer (**#**ND-2000 C, Thermo Fisher Scientific, USA), and cDNA was synthesized using the Illustra Ready-To-Go™ RT-PCR Beads (#27926701, Cytiva, UK). Gene expression analysis was performed by quantitative real-time polymerase chain reaction (qRT-PCR) using SensiFast™ Sybr Hi-Rox Mix (**#**BIO-92020, Bioline, UK) and primers (**#**OLIG-1, Microsynth AG, Switzerland) designed using Primer blast (https://www.ncbi.nlm.nih.gov/tools/primer-blast/, Table 1) or according to the literature (Table 2). Samples were measured on the Real-Time PCR instrument Quant Studio 5 (**#**A28573, Thermo Fisher Scientific, USA). Ribosomal protein S29 (RPS29) - encoding gene was used as an internal control for evaluation of qRT-PCR, and data were presented as a fold change relative to control values according to the ΔΔct calculation method [30].

**Table 1:** Primer sequences used for the analysis of gene expression by quantitative real-time PCR (qRT-PCR)

Gene transcript	Forward primer (5’-3’)	Reverse primer (5’-3’)
α1B-AR	ACCTTGGGCATTGTAGTCGGA	AGCCAGAACACCACCTTGAACA
α2A-AR	GCGCCCCAGAACCTCTTCCTGGTG	GAGTGGCGGGAAAAGGATGACGGC
β1-AR	GCCGATCTGGTCATGGGA	GTTGTAGCAGCGGCGCG
β2-AR	ACATCACTCAGGAACGGGACGAAG	CAGCACACGCCAAGGAGGTTATG
CCL2	TCCACCACTATGCAGGTCTC	CATTAACTGCATCTGGCTGAG
CCL21	GTGCTAACGATGCGGAAGAC	AGAGCTGGTAGCCCCTTAGT
CD40	ACCGACACTGCGAACTCAA	GCAGGGTTGGCAGACAGTA
CD69	GTGCTCGTAGTGGTCCTCATC	CAGCAAGGGTAGCGTCATCT
CD80	TCAAATTCCGACGCTGCTTCA	CTCAAAACCACCAGAGAGGCT
CHAT	TGGCCCGAGACCTGTGCAAA	TTGGGTGCTGGTGGCTTGCT
COX-2	GATGACGAGCGACTGTTCCA	TGGTAACCGCTCAGGTGTTG
GM-CSF	TGGCGCCTTGACCATGATAG	TTCACAGTCAGTTTCCGGGG
GR	GTGCTGACATGTGGAAGCTG	CAATCGTTTCTTCCAGCACA
IFNγ	GCCCTCTCTGGCTGTTACTG	CGTGTTACCGTCCTTTTGCC
IL-1β	GACCTGTTCTTTGAGGCTGACA	CTCATCTGGACAGCCCAAGTC
IL-2	GCAGCGTGTGTTGGATTTGACTC	TGTTGAGATGATGCTTTGACAGATGGC
IL-4	GGCTTCCAGGGTGCTTCGCAAAT	TGTGAGCGTGGACTCATTCACGG
IL-6	AAGTCGGAGGCTTAATTACATATGTTC	TCATCGCTGTTCATACAATCAGAA
IL-10	AACTGCACCCACTTCCCAGT	CACTGCCTTGCTTTTATTCTCAC
IL-12 A	CAGGCCATAAATGCAGCAC	CCGCTGTGATTCAGAGACC
IL-12Rβ	CTCCAACAAGGGAGCGTCAT	TGCATGTACCCGGATTTCGT
IL-17 A	GTTCAGTGTGTCCAAACGCC	AGGGTGAAGTGGAACGGTTG
LY6C	TGCAGAAAGAGCTCAGGGC	TGCTCTTTGCTGTCCGTCTT
MPO	CCATCCCTCCACTTCATGGG	CACCTCTCACTGACAACGGG
RPS29	GGTCGCTTAGTCCAACTTAATGAA	GCTGAACATGTGCCGACAGT
SIGNR1	TCTTGATTCCATGGAGGACGG	CAGGCATCCTGCCAAACTCT
STAT3	AAGCTGACCCAGGTAGTGCT	CGGCAGGTCAATGGTATTGC
TH	TCTCCACGGTGTACTGGTTCACT	AGGCTGGTAGGTTTGATCTTGGTA
TNFα	ATGGGCTCCCTCTCATCAGT	GCTTGGTGGTTTGCTACGAC

Abbreviations: α1B-AR, alpha 1B adrenergic receptor; α2A-AR, alpha 2 A adrenergic receptor; β1-AR, beta 1 adrenergic receptor; β2-AR, beta 2 adrenergic receptor; CCL2, C-C motif chemokine ligand 2; CCL21, C-C motif chemokine ligand 21; CD40, cluster of differentiation 40; CD69, cluster of differentiation 69; CD80, cluster of differentiation 80; CHAT, choline acetyltransferase; COX-2, cyclooxygenase-2; GM-CSF, granulocyte-macrophage colony-stimulating factor; GR, glucocorticoid receptor; IFNγ, interferon gamma; IL-1β, interleukin 1 beta; IL-2, interleukin 2; IL-4, interleukin 4; IL-6, interleukin 6; IL-10, interleukin 10; IL-12 A, interleukin 12 A; IL-12Rβ, interleukin 12 receptor beta; IL-17 A, interleukin 17 A; LY6C, lymphocyte antigen 6 complex; MPO, myeloperoxidase; RPS29, ribosomal protein S29; SIGNR1, specific intercellular adhesion molecule-3-grabbing non-integrin receptor 1; STAT3, signal transducer and activator of transcription 3; TH, tyrosine hydroxylase; TNFα, tumor necrosis factor alpha

**Table 2:** Primer sequences used for amplification of bacterial 16 S rRNA

	Forward primer (5´-3´)	Reverse primer (5´-3´)	References
**Total bacteria**	ACTCCTACGGGAGGCAGCAGT	ATTACCGCGGCTGCTGGC	[24]
***Firmicutes***	ACCCGCGTCTGATTAGCTAGTT	CCTCTCAGGCCGGCTACTG	[25]
***Bacteroidetes***	GTTTAATTCGATGATACGCGAG	TTAASCCGACACCTCACGG	[26]
***Actinobacteria***	TGTAGCGGTGGAATGCGC	AATTAAGCCACATGCTCCGCT	[26]
***δ/γ - Proteobacteria***	GCTAACGCATTAAGTRYCCCG	GCCATGCRGCACCTGTCT	[26]
***Lactobacillus***	AGCAGTAGGGAATCTTCCA	CGCCACTGGTGTTCYTCCATATA	[27]
***Prevotella***	CACRGTAAACGATGGATGCC	GGTCGGGTTGCAGACC	[28]
***Ruminococcus***	GAAAGCGTGGGGAGCAAACAGG	GACGACAACCATGCACCACCTG	[29]

### Measurement of plasma CORT

Trunk blood from rats was collected into heparinized tubes and centrifuged at 10,000 × g for 20 min. Plasma was removed and stored at -80 °C for further analysis.

Plasma CORT levels were measured by an ELISA kit (**#**ADI-900-097, Corticosterone ELISA Kit, Enzo Life Sciences, USA), according to the manufacturer’s instructions.

### Statistical analysis

All data were analyzed using SigmaPlot 11.0 software (Systat Software, Inc., USA). The outliers were excluded if the data points ranged more than 1.5 interquartile distances below the first quartile or above the third quartile (“Outlier Calculator” at https://MiniWebtool.com/outlier-calculator/ from MiniWebtool, https://MiniWebtool.com/). Normal distribution of the obtained data was checked by the Shapiro-Wilks test. If the groups were not of homogeneous variance, a logarithm or square root transform was applied. The data were tested with a one-way ANOVA (for the factor SPS/SPS + ARI treatment) or a two-way ANOVA (for the factors SPS/SPS + ARI treatment and ex vivo stimulation) with Fisher´s LSD *post hoc* test. If the data distribution remained non-homogeneous despite transformation, the non-parametric Kruskal–Wallis test was applied (for IL-1β mRNA after LPS stimulation) followed by multiple comparisons. Results are presented as mean ± SEM for parametric data, and as median ± interquartile range for non-parametric data. Differences were considered significant at *p* < 0.05. Moreover, Pearson’s correlation analysis was conducted between the parameters in question in GraphPad Prism version 8 (GraphPad Software, Inc., USA).

## Results

### EPM test

**Fig. 1:**
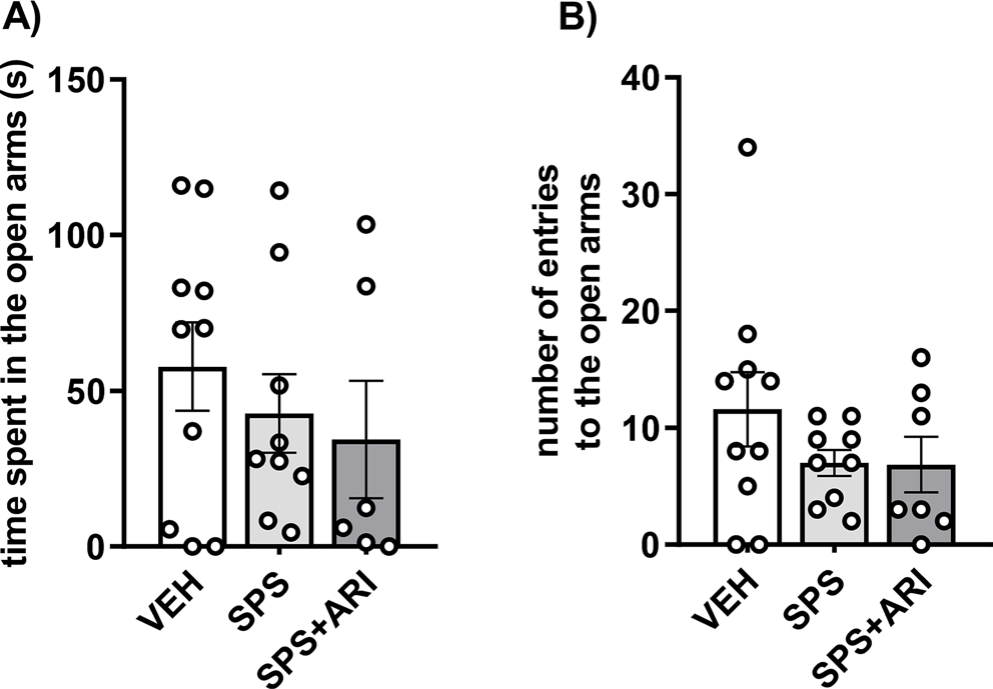
Effects of the treatment with aripiprazole (ARI) on elevated plus maze (EPM) performance in single prolonged stress (SPS)-exposed rats. Time spent in the open arms (**A**) and number of open-arm entries (**B**) during a 5 min EPM test were assessed. Unstressed rats were treated with vehicle (VEH; 2% Tween 20 in saline; *n* = 10) for 28 days. Rats exposed to SPS received vehicle (SPS; *n* = 9) or ARI (5 mg/kg) (SPS + ARI; *n* = 6–7) for 28 days. Data are presented as mean ± SEM. Statistical analysis was performed using one-way ANOVA followed by Fisher’s LSD *post hoc* test. Abbreviations: ARI, aripiprazole; EPM, elevated plus maze; SEM, standard error of the mean; SPS, single prolonged stress; VEH, vehicle

In the EPM, one-way ANOVA revealed no significant effect of SPS/SPS + ARI treatment on either the time spent in the OA (F_2, 22_ = 0.6, *p* = 0.55, Fig. 1A) or the number of OA entries (F_2, 23_ = 1.3, *p* = 0.31, Fig. 1B).

### Stress response and the neuroendocrine regulation in the spleen

To evaluate the effects of SPS and SPS + ARI on the stress response and splenic neuroendocrine system, we measured plasma CORT and gene expression of receptors involved in the stress response, including the glucocorticoid receptor (GR) and several subtypes of adrenergic receptors i.e. alpha 1B adrenergic receptors (α1B-AR), alpha 2 A adrenergic receptors (α2A-AR), beta 1 adrenergic receptors (β1-AR), and beta 2 adrenergic receptors (β2-AR). Based on the previously described role of T cell-produced catecholamines in Th17-associated PTSD pathology [5, 31], we determined expression of tyrosine hydroxylase (TH), the rate-limiting enzyme in catecholamine biosynthesis. For the cholinergic anti-inflammatory pathway, which is associated with acetylcholine production in splenocytes, particularly T and B cells [32], we analyzed choline acetyltransferase (CHAT) gene expression.

**Fig. 2:**
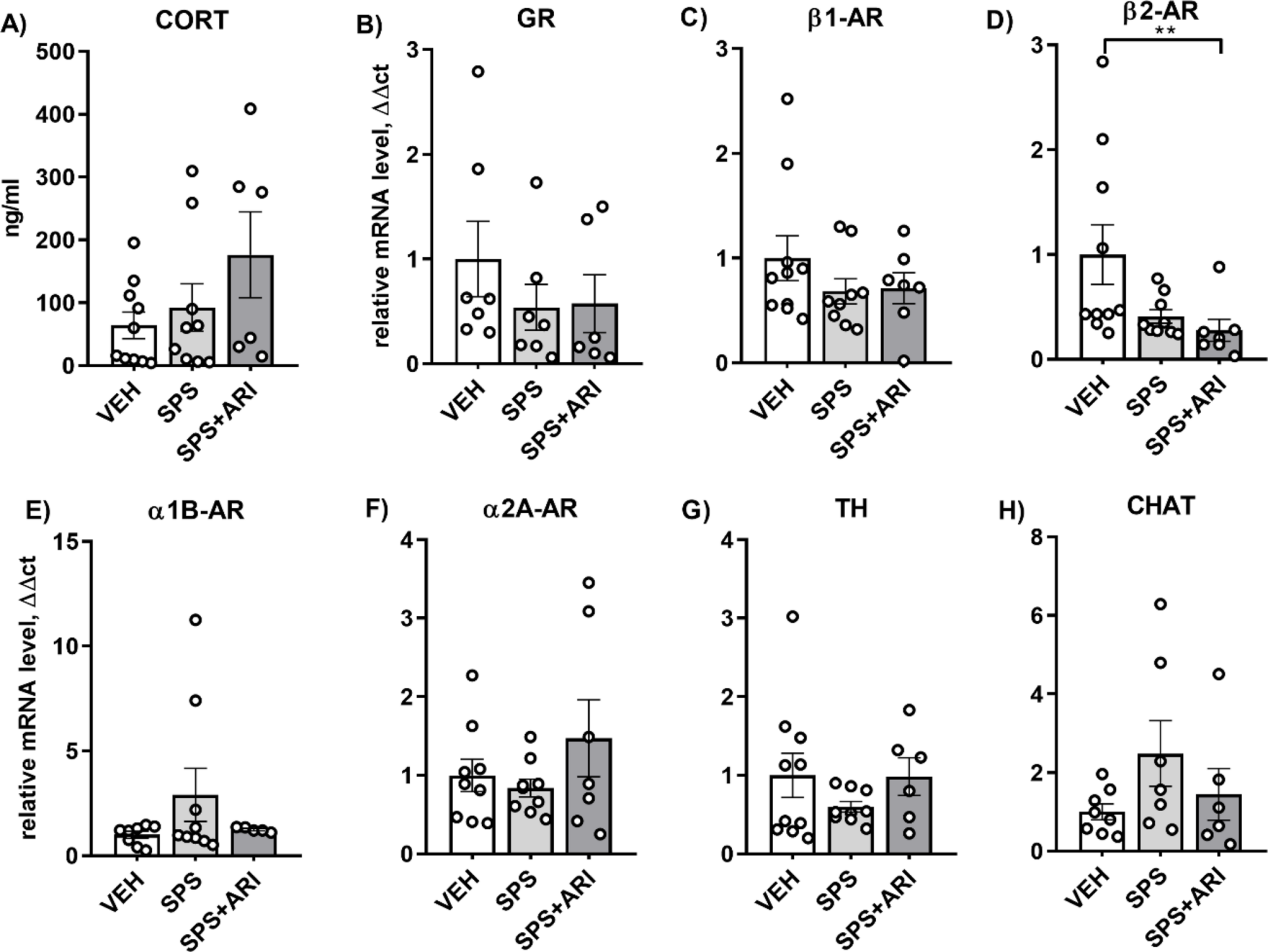
Effects of the treatment with aripiprazole (ARI) on plasma corticosterone (CORT, **A**) and expression of glucocorticoid receptor (GR, **B**); adrenergic receptor subtypes β1-AR (**C**), β2-AR (**D**), α1B-AR (**E**), and α2A-AR (**F**); tyrosine hydroxylase (TH, **G**); and choline acetyltransferase (CHAT, **H**) in single prolonged stress (SPS)-exposed rats. Unstressed rats were treated with vehicle (VEH; 2% Tween 20 in saline; *n* = 10) for 28 days. Rats exposed to SPS received vehicle (SPS; *n* = 9) or ARI (5 mg/kg) (SPS + ARI; *n* = 6–7) for 28 days. CORT levels were measured by ELISA, and mRNA levels were quantified by qRT-PCR, normalized to RPS29, and expressed as fold change vs. control. Data are presented as mean ± SEM. Statistical analysis was performed using one-way ANOVA followed by Fisher’s LSD *post hoc* test. **p < 0.01. Abbreviations: α1B-AR, alpha 1B adrenergic receptor; α2A-AR, alpha 2 A adrenergic receptor; β1-AR, beta 1 adrenergic receptor; β2-AR, beta 2 adrenergic receptor; ARI, aripiprazole; ELISA, Enzyme-linked immunosorbent assay; CHAT, choline acetyltransferase; CORT, corticosterone; GR, glucocorticoid receptor; qRT-PCR, quantitative real-time polymerase chain reaction; RPS29, ribosomal protein S29; SEM, standard error of the mean; SPS, single prolonged stress; TH, tyrosine hydroxylase; VEH, vehicle

One-way ANOVA revealed no significant effect of SPS/SPS + ARI treatment on plasma CORT levels (F_2,22_ = 1.9, *p* = 0.26, Fig. 2A), splenic GR mRNA (F_2,17_ = 0.8, *p* = 0.47, Fig. 2B), or β1-AR mRNA expression (F_2,21_ = 0.1, *p* = 0.98, Fig. 2C). In contrast, a significant effect was observed for β2-AR mRNA expression (F_2,23_ = 6.1, *p* = 0.008, Fig. 2D). *Post hoc* analysis (Fisher’s LSD) indicated a strong trend toward reduced β2-AR mRNA levels in SPS rats (*p* = 0.058, Fig. 2D) and a significant reduction in SPS + ARI rats (*p* = 0.002, Fig. 2D). No significant effects were detected for α1B-AR (F_2,19_ = 1.4, *p* = 0.26, Fig. 2E), α2A-AR (F_2,22_ = 1.3, *p* = 0.29, Fig. 2F), TH (F_2,22_ = 1.1, *p* = 0.35, Fig. 2G), or CHAT mRNA expression (F_2,18_ = 1.7, *p* = 0.21, Fig. 2H).

### Determination of microbiota in the spleen

We analyzed the presence of bacteria in the spleen using qRT-PCR amplification of 16 S rRNA specific for total bacteria, and various taxa, including phyla *Firmicutes*,* Bacteroidetes*,* Actinobacteria*, classes *γ/δ-Proteobacteria*, as well as Gram-positive genera *Lactobacillus* and *Ruminococcus* and Gram-negative genus *Prevotella*.

**Fig. 3:**
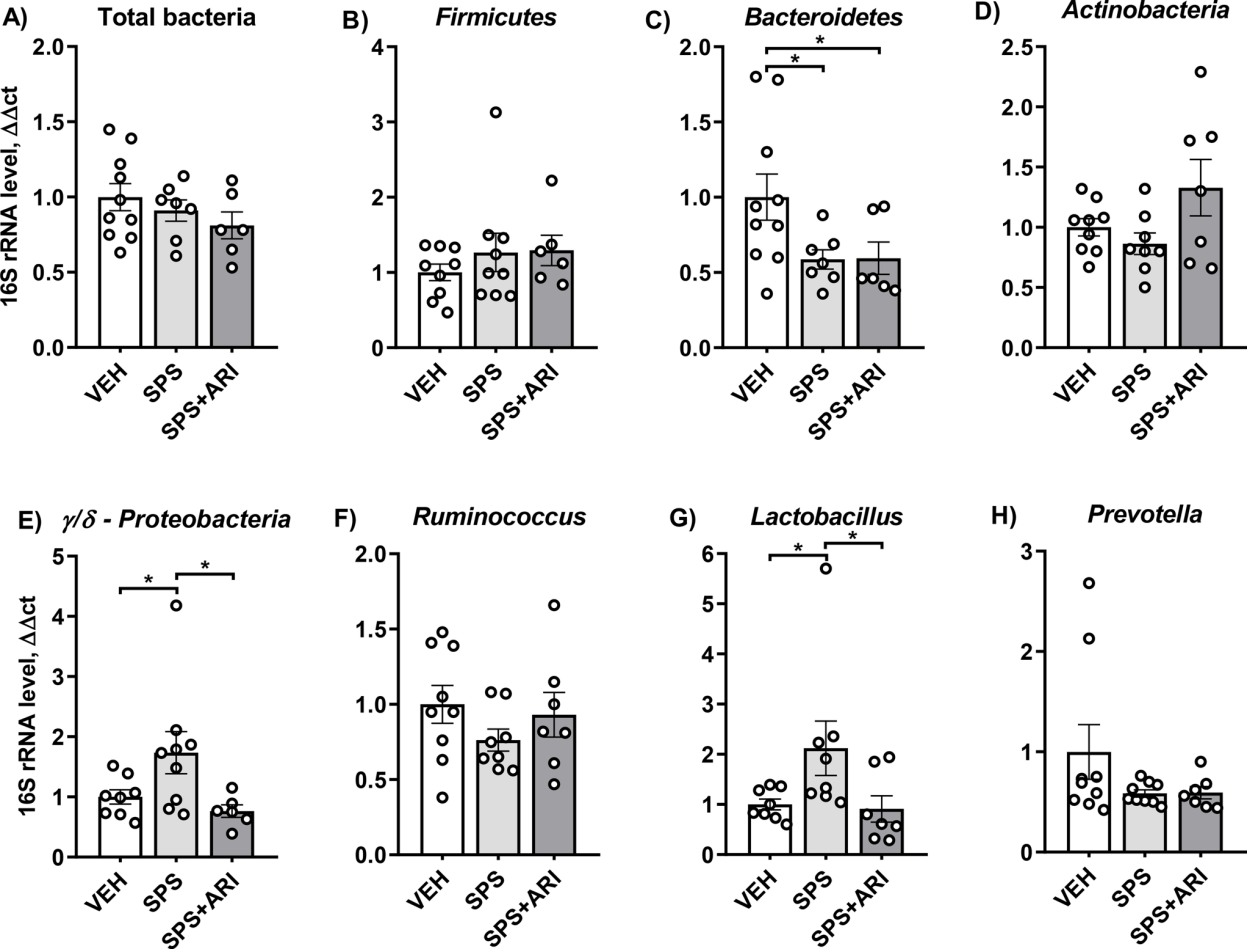
Effects of the treatment with aripiprazole (ARI) on bacterial 16 S rRNA levels corresponding to total bacteria (**A**), *Firmicutes* (**B**), *Bacteroidetes* (**C**), *Actinobacteria* (**D**), γ/δ-*Proteobacteria* (**E**), *Ruminococcus* (**F**), *Lactobacillus* (**G**), and *Prevotella* (**H**) in single prolonged stress (SPS)-exposed rats. Unstressed rats were treated with vehicle (VEH; 2% Tween 20 in saline; *n* = 10) for 28 days. Rats exposed to SPS received vehicle (SPS; *n* = 9) or ARI (5 mg/kg) (SPS + ARI; *n* = 6–7) for 28 days. 16 S rRNA levels were determined by qRT-PCR, normalized to RPS29, and expressed as fold change vs. control. All data are presented as mean ± SEM and analyzed with one-way ANOVA, followed by Fisher´s LSD *post hoc* test. *p < 0.05. Abbreviations: 16 S rRNA, 16 S subunit of ribosomal ribonucleic acid; ARI, aripiprazole; qRT-PCR, quantitative real-time polymerase chain reaction; RPS29, ribosomal protein S29; SEM, standard error of the mean; SPS, single prolonged stress; VEH, vehicle

One-way ANOVA revealed no significant effect of SPS/SPS + ARI treatment on 16 S rRNA levels of total bacteria (F_2,20_ = 1.1, *p* = 0.34, Fig. 3A) or *Firmicutes* (F_2,22_ = 1.5, *p* = 0.24, Fig. 3B). In contrast, a significant effect was observed for *Bacteroidetes* (F_2,20_ = 3.6, *p* = 0.046, Fig. 3C), with *post hoc* analysis (Fisher’s LSD) showing reduced levels in both SPS (*p* = 0.032, Fig. 3C) and SPS + ARI groups (*p* = 0.043, Fig. 3C). No significant effect was found for *Actinobacteria* (F_2,21_ = 2.8, *p* = 0.081, Fig. 3D). However, SPS/SPS + ARI treatment significantly affected γ/δ-*Proteobacteria* levels (F_2,20_ = 4.1, *p* = 0.032, Fig. 3E), with SPS increasing their abundance (*p* = 0.043, Fig. 3E) and ARI attenuating this effect (*p* = 0.016, Fig. 3E). No significant effect was observed for *Ruminococcus* (F_2,21_ = 1.1, *p* = 0.35, Fig. 3F). In contrast, a significant effect was detected for *Lactobacillus* (F_2,20_ = 3.5, *p* = 0.048, Fig. 3G), with increased levels following SPS (*p* = 0.037, Fig. 3G) and suppression by ARI (*p* = 0.030, Fig. 3G). No significant effect was found for *Prevotella* (F_2,22_ = 1.9, *p* = 0.17, Fig. 3H).

### Immune cells in the spleen

In the spleen tissue, we measured several molecular markers by qRT-PCR, which may reflect changes in splenocyte populations and involvement in immune cell functioning. These included: lymphocyte antigen 6 complex (LY6C), which is highly expressed in inflammatory monocytes; costimulatory molecule cluster of differentiation 80 (CD80) expressed in antigen-presenting cells; specific intercellular adhesion molecule-3-grabbing non-integrin receptor 1 (SIGNR1) expressed in marginal zone macrophages; and chemokine C-C motif chemokine ligand 21 (CCL21) guiding T cells and dendritic cells into lymphoid tissues.

**Fig. 4:**
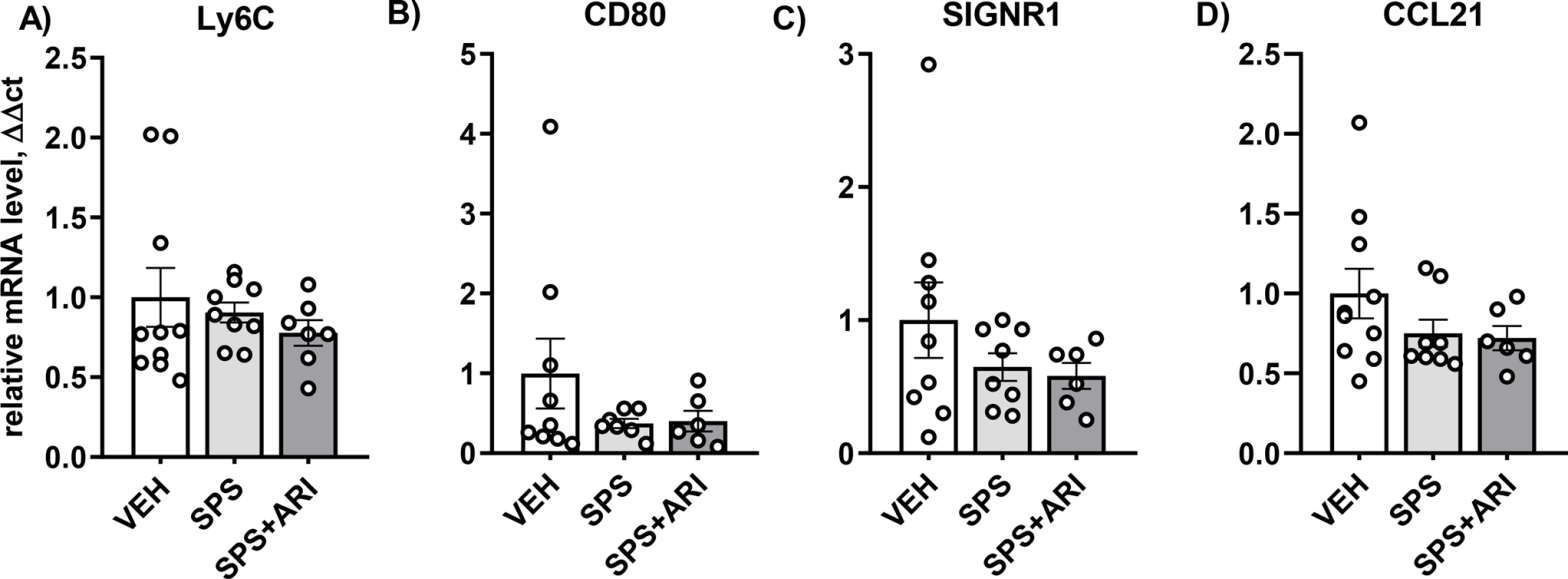
Effects of the treatment with aripiprazole (ARI) on gene expression of lymphocyte antigen 6 complex (LY6C, **A**), cluster of differentiation 80 (CD80, **B**), specific intercellular adhesion molecule-3-grabbing non-integrin receptor 1 (SIGNR1, **C**), and C-C motif chemokine ligand 21 (CCL21, **D**) in the spleen of rats exposed to single prolonged stress (SPS). Unstressed rats were treated with vehicle (VEH; 2% Tween 20 in saline; *n* = 9–10) for 28 days. Rats exposed to SPS received vehicle (SPS; *n* = 8–9) or ARI (5 mg/kg) (SPS + ARI; *n* = 6–7) for 28 days. Expression levels were quantified by qRT-PCR, normalized to RPS29, and expressed as fold change vs. control. Data are presented as mean ± SEM. Statistical analysis was performed using one-way ANOVA followed by Fisher’s LSD *post hoc* test. Abbreviations: ARI, aripiprazole; CCL21, C-C motif chemokine ligand 21; CD80, cluster of differentiation 80; LY6C, lymphocyte antigen 6 complex; qRT-PCR, quantitative real-time polymerase chain reaction; RPS29, ribosomal protein S29; SIGNR1, specific intercellular adhesion molecule-3-grabbing non-integrin receptor 1; SEM, standard error of the mean; SPS, single prolonged stress; VEH, vehicle

One-way ANOVA revealed no significant effect of SPS/SPS + ARI treatment on mRNA levels of LY6C (F_2,23_ = 0.7, *p* = 0.53, Fig. 4A), CD80 (F_2,19_ = 1.3, *p* = 0.29, Fig. 4B), SIGNR1 (F_2,20_ = 1.2, *p* = 0.32, Fig. 4C), or CCL21 (F_2,21_ = 1.5, *p* = 0.24, Fig. 4D).

### Ex vivo stimulation of splenocytes with LPS

To assess the effects of SPS and SPS + ARI on the inflammatory response in splenocytes, cells were stimulated ex vivo with LPS for 3 h, and afterwards, changes in gene expression of selected inflammatory mediators and cytokines were analyzed.

**Fig. 5:**
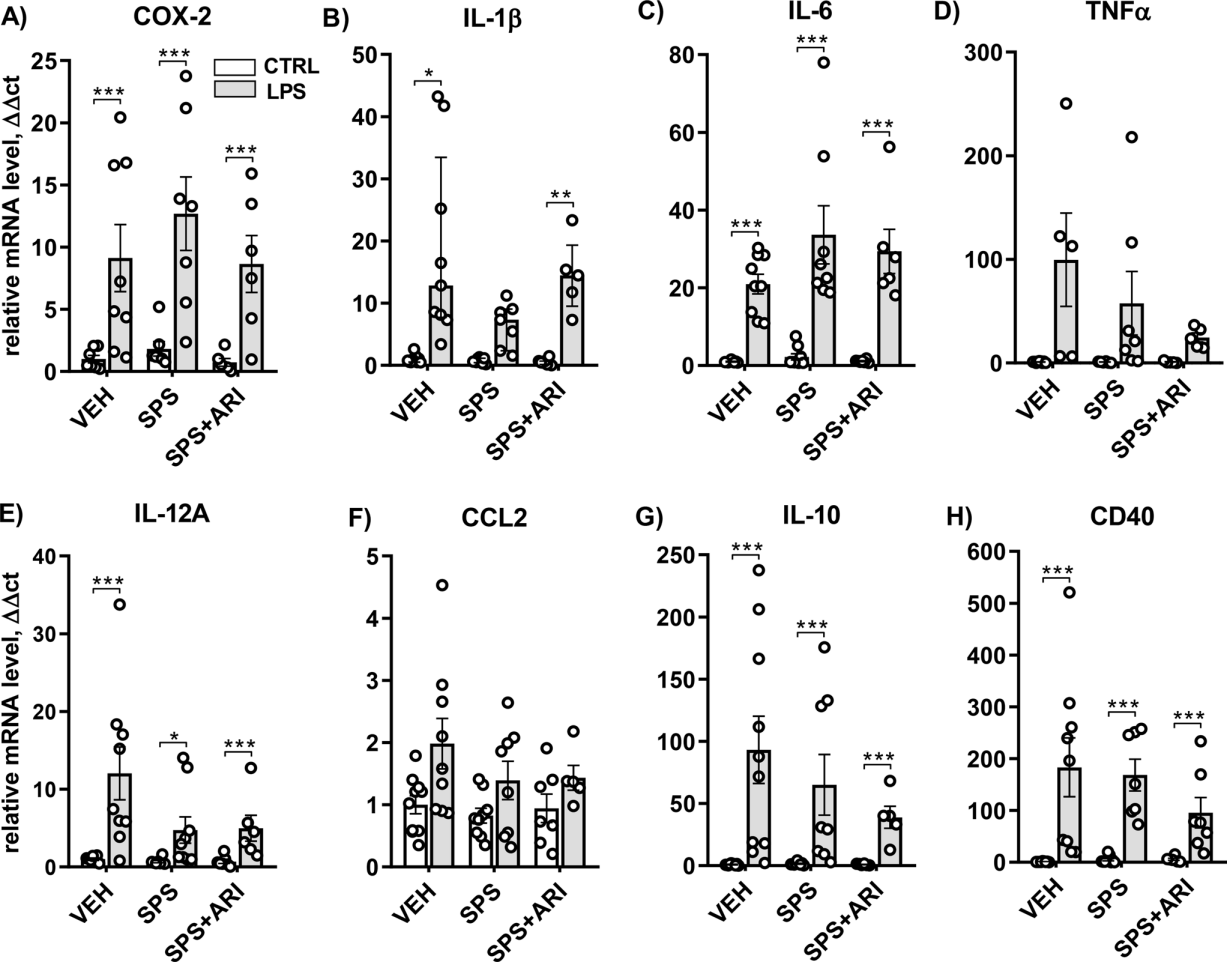
Effects of the treatment with aripiprazole (ARI) on gene expression of cyclooxygenase-2 (COX-2, **A**), interleukin 1β, (IL-1β, **B**), interleukin 6 (IL-6, **C**), tumor necrosis factor alpha (TNFα, D), interleukin 12 A (IL-12 A, **E**), motif chemokine ligand 2 (CCL2, **F**), interleukin 10 (IL-10, **G**), CD40 (**H**) in the splenocytes isolated from rats exposed to single prolonged stress (SPS). Splenocytes were stimulated ex vivo with H_2_O (CTRL) or lipopolysaccharide (LPS, 10 µg/ml) for 3 h. Unstressed rats were treated with vehicle (VEH; 2% Tween 20 in saline; *n* = 8–10) for 28 days. Rats exposed to SPS received vehicle (SPS; *n* = 8–10) or ARI (5 mg/kg) (SPS + ARI; *n* = 6–7) for 28 days. Expression levels were quantified by qRT-PCR, normalized to RPS29, and expressed as fold change vs. control. Data are presented as mean ± SEM and analyzed with two-way ANOVA followed by Fisher’s LSD *post hoc* test; except for IL-1β, which is reported as median with interquartile ranges and analyzed using the Kruskal–Wallis test followed by multiple comparisons. *p < 0.05, **p < 0.01, ***p < 0.001 splenocyte treatment versus the corresponding CTRL group. Abbreviations: ARI, aripiprazole; CCL2, C-C motif chemokine ligand 2; CD40, cluster of differentiation 40; COX-2, cyclooxygenase-2; CTRL, control; IL-1β, interleukin 1 beta; IL-6, interleukin 6; IL-10, interleukin 10; IL-12 A, interleukin 12 A; LPS, lipopolysaccharide; qRT-PCR, quantitative real-time polymerase chain reaction; RPS29, ribosomal protein S29; SEM, standard error of the mean; SPS, single prolonged stress; TNFα, tumor necrosis factor alpha; VEH, vehicle

Two-way ANOVA revealed a significant effect of LPS stimulation on cyclooxygenase-2 (COX-2) mRNA levels (F_1,37_ = 57.6, *p* < 0.001, Fig. 5A), whereas neither SPS/SPS + ARI treatment (F_2,37_ = 0.8, *p* = 0.47, Fig. 5A) nor the interaction between factors (F_2,37_ = 0.4, *p* = 0.65, Fig. 5A) reached statistical significance. Fisher’s LSD *post hoc* analysis demonstrated that LPS significantly increased COX-2 mRNA levels in splenocytes derived from VEH, SPS, and SPS + ARI rats (*p* < 0.001 for all, Fig. 5A). Due to non-normal data distribution, IL-1β mRNA levels were analyzed using the Kruskal–Wallis test, which revealed a significant effect of experimental groups (H(6) = 29.9, *p* < 0.001, Fig. 5B). Dunn’s multiple comparisons test showed that LPS stimulation significantly increased IL-1β mRNA levels in splenocytes from VEH (*p* = 0.046, Fig. 5B) and SPS + ARI rats (*p* = 0.0024, Fig. 5B). Similarly, two-way ANOVA revealed a significant effect of LPS stimulation on IL-6 mRNA levels (F_1,41_ = 381.3, *p* < 0.001, Fig. 5C) whereas neither SPS/SPS + ARI treatment (F_2,41_ = 1.2, *p* = 0.317, Fig. 5C) nor the interaction (F_2,41_ = 1.1, *p* = 0.35, Fig. 5C) was significant. *Post hoc* analysis confirmed that LPS significantly increased IL-6 mRNA levels in splenocytes from VEH, SPS, and SPS + ARI groups (*p* < 0.001 for all, Fig. 5C). For TNFα mRNA levels, two-way ANOVA revealed only a significant effect of LPS stimulation (F_1,33_ = 54.6, *p* < 0.001, Fig. 5D) with no effect of SPS/SPS + ARI treatment (F_2,33_ = 0.00019, *p* = 1.0, Fig. 5D) or their interaction (F_2,33_ = 0.1, *p* = 0.96, Fig. 5D). A similar pattern was observed for interleukin 12 A (IL-12 A) mRNA levels. Two-way ANOVA showed a significant effect of LPS stimulation (F_1,41_ = 33.0, *p* < 0.001, Fig. 5E) but no effect of SPS/SPS + ARI treatment (F_2,41_ = 1.2, *p* = 0.31, Fig. 5E) or interaction (F_2,41_ = 0.7, *p* = 0.52, Fig. 5E). Fisher’s LSD *post hoc* test revealed that LPS significantly increased IL-12 A mRNA levels in splenocytes from VEH (*p* < 0.001, Fig. 5E), SPS (*p* = 0.013, Fig. 5E), and SPS + ARI rats (*p* < 0.001, Fig. 5E). For C-C motif chemokine ligand 2 (CCL2) mRNA levels, two-way ANOVA identified a significant effect of LPS stimulation (F_1,44_ = 4.3, *p* = 0.045, Fig. 5F), whereas SPS/SPS + ARI treatment (F_2,44_ = 0.8, *p* = 0.47, Fig. 5F) and the interaction term (F_2,44_ = 0.2, *p* = 0.85, Fig. 5F) were not significant. Likewise, two-way ANOVA demonstrated a significant effect of LPS stimulation on interleukin 10 (IL-10) mRNA levels (F_1,42_ = 69.2, *p* < 0.001, Fig. 5G) with no effect of experimental conditions (F_2,42_ = 0.9, *p* = 0.41, Fig. 5G) or their interaction (F_2,42_ = 0.9, *p* = 0.40, Fig. 5G). *Post hoc* test showed that LPS significantly increased IL-10 mRNA levels in splenocytes derived from VEH, SPS, and SPS + ARI groups (*p* < 0.001 for all, Fig. 5G). Finally, for cluster of differentiation 40 (CD40) expression, two-way ANOVA revealed a significant effect of LPS stimulation (F_1,44_ = 104.3, *p* < 0.001, Fig. 5H), whereas neither SPS/SPS + ARI treatment (F_2,44_ = 0.8, *p* = 0.47, Fig. 5H) nor the interaction between factors (F_2,44_ = 0.4, *p* = 0.672, Fig. 5H) was significant. Consistent with the other inflammatory markers, LPS significantly increased CD40 mRNA levels in splenocytes derived from VEH, SPS, and SPS + ARI rats (*p* < 0.001 for all, Fig. 5H).

### Ex vivo stimulation of splenocytes with PMA/ionomycin

Considering the established involvement of pro-inflammatory CD4⁺ T-cell subsets, particularly Th1 and Th17 cells, in the PTSD pathophysiology [7, 33], we stimulated splenocytes with PMA and ionomycin for 3 h to induce T-cell activation and cytokine production.

**Fig. 6:**
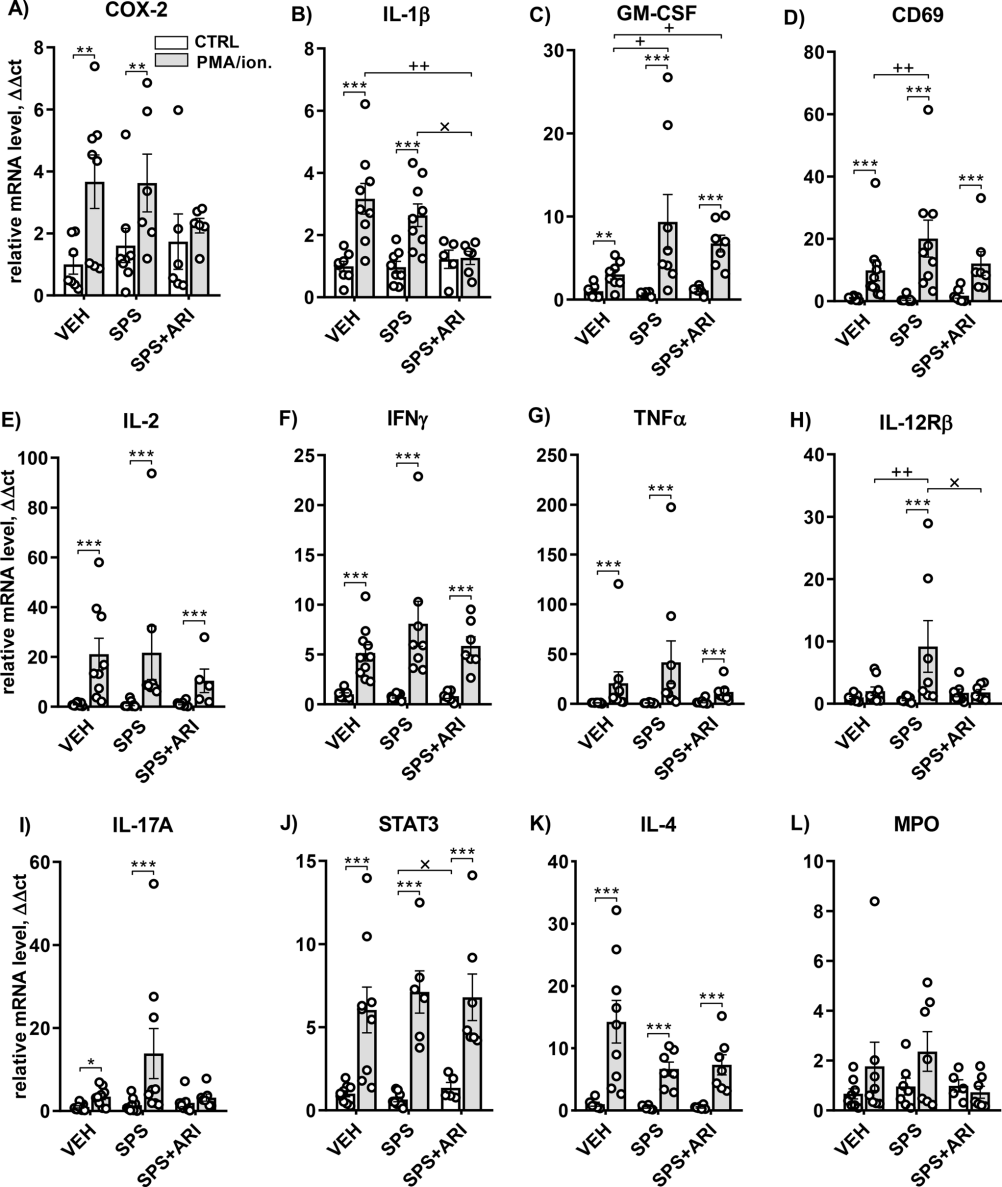
Effects of the treatment with aripiprazole (ARI) on gene expression cyclooxygenase-2 (COX-2, **A**) interleukin 1 beta, (IL-1β, **B**), granulocyte-macrophage colony-stimulating factor (GM-CSF, **C**), cluster of differentiation 69 (CD69, D), interleukin 2 (IL-2, **E**), interferon gamma (IFNγ, **F**), tumor necrosis factor alpha (TNFα, **G**), interleukin 12 receptor beta (IL-12Rβ, **H**), interleukin 17 A (IL-17 A, **I**), signal transducer and activator of transcription 3 (STAT3, **J**), interleukin 4 (IL-4, **K**), myeloperoxidase (MPO, L) in the splenocytes isolated from rats exposed to single prolonged stress (SPS). Splenocytes were stimulated ex vivo with H_2_O (CTRL) or phorbol 12-myristate 13-acetate (PMA, 81 nmol/l) and ionomycin (ion., 1.34 µmol/l) for 3 h. Unstressed rats were treated with vehicle (VEH; 2% Tween 20 in saline; *n* = 8–10) for 28 days. Rats exposed to SPS received vehicle (SPS; *n* = 8–10) or ARI (5 mg/kg) (SPS + ARI; *n* = 6–7) for 28 days. Expression levels were quantified by qRT-PCR, normalized to RPS29, and expressed as fold change vs. control. Data are presented as mean ± SEM and analyzed using two-way ANOVA followed by Fisher’s LSD *post hoc* test. *p < 0.05, **p < 0.01, ***p < 0.001 splenocyte treatment versus the corresponding CTRL group, ^+^*p* < 0.05, ^++^*p* < 0.01 versus VEH treated group, and ^×^*p* < 0.05 - versus SPS exposed group. Abbreviations: ARI, aripiprazole; CD69, cluster of differentiation 69; COX-2, cyclooxygenase-2; CTRL, control; GM-CSF, granulocyte-macrophage colony-stimulating factor; ion., ionomycin; IL-1β, interleukin 1 beta; IL-2, interleukin 2; IL-4, interleukin 4; IL-12Rβ, interleukin 12 receptor beta; IL-17 A, interleukin 17 A; IFNγ, interferon gamma; LPS, lipopolysaccharide; MPO, myeloperoxidase; PMA, phorbol 12-myristate 13-acetate; qRT-PCR, quantitative real-time polymerase chain reaction; RPS29, ribosomal protein S29; SEM, standard error of the mean; SPS, single prolonged stress; STAT3, signal transducer and activator of transcription 3; TNFα, tumor necrosis factor alpha; VEH, vehicle

For COX-2 gene expression, two-way ANOVA revealed a significant effect of PMA/ionomycin stimulation (F_1,39_ = 18.6, *p* < 0.001, Fig. 6A), whereas neither SPS/SPS + ARI treatment (F_2,39_ = 1.0, *p* = 0.38, Fig. 6A) nor the interaction between factors (F_2,39_ = 0.3, *p* = 0.76, Fig. 6A) was significant. *Post hoc* analysis using Fisher’s LSD test showed significantly increased COX-2 expression in stimulated splenocytes derived from VEH (*p* = 0.006, Fig. 6A) and SPS-exposed rats (*p* = 0.002, Fig. 6A). Similarly, IL-1β expression was significantly affected by PMA/ionomycin stimulation (F_1,39_ = 20.2, *p* < 0.001, Fig. 6B), whereas neither SPS/SPS + ARI treatment (F_2,39_ = 1.6, *p* = 0.21, Fig. 6B) nor the interaction of the factors (F_2,39_ = 2.6, *p* = 0.086, Fig. 6B) reached significance. *Post hoc* analysis demonstrated elevated IL-1β mRNA levels in stimulated splenocytes from VEH and SPS groups (*p* < 0.001 for both, Fig. 6B). Notably, although PMA/ionomycin induced comparable IL-1β responses in VEH and SPS rats, this response was significantly attenuated in SPS + ARI animals (*p* = 0.006 vs. VEH and *p* = 0.022 vs. SPS, Fig. 6B). For granulocyte-macrophage colony-stimulating factor (GM-CSF), two-way ANOVA showed a significant effect of PMA/ionomycin stimulation (F_1,38_ = 66.9, *p* < 0.001, Fig. 6C) while neither effect of SPS/SPS + ARI treatment (F_2,38_ = 2.5, *p* = 0.094, Fig. 6C) nor the interaction (F_2,38_ = 1.8, *p* = 0.17, Fig. 6C) was significant. PMA/ionomycin increased GM-CSF expression in all experimental groups (VEH: *p* = 0.002, SPS and SPS + ARI: *p* < 0.001, Fig. 6C). Following PMA/ionomycin stimulation, GM-CSF expression was higher in SPS (*p* = 0.021, Fig. 6C) and SPS + ARI splenocytes (*p* = 0.02, Fig. 6C) than in VEH ones. A similar pattern was observed for the cluster of differentiation 69 (CD69). Two-way ANOVA revealed a significant effect of PMA/ionomycin stimulation (F_1,41_ = 89.1, *p* < 0.001, Fig. 6D), no effect of SPS/SPS + ARI treatment (F_2,41_ = 1.1, *p* = 0.36, Fig. 6D) but notably, marked effect of their interaction (F_2,41_ = 5.1, *p* = 0.010, Fig. 6D). PMA/ionomycin robustly increased CD69 expression in all experimental groups (*p* < 0.001 for all, Fig. 6D). Moreover, stimulated splenocytes from SPS-exposed rats exhibited higher CD69 expression than those from VEH animals (*p* = 0.003, Fig. 6D). For interleukin 2 (IL-2), two-way ANOVA showed a significant effect of PMA/ionomycin stimulation (F_1,39_ = 95.5, *p* < 0.001, Fig. 6E) with no effect of SPS/SPS + ARI treatment (F_2,39_ = 1.5, *p* = 0.24, Fig. 6E) or interaction (F_2,39_ = 1.3, *p* = 0.28, Fig. 6E). P*ost hoc* Fisher’s LSD test showed significantly increased IL-2 expression in splenocytes from all experimental groups following PMA/ionomycin stimulation (*p* < 0.001 for all, Fig. 6E). Likewise, interferon gamma (IFNγ) expression was strongly affected by PMA/ionomycin stimulation (F_1,41_ = 129.3, *p* < 0.001, Fig. 6F) whereas neither effect of SPS/SPS + ARI treatment (F_2,41_ = 0.2, *p* = 0.86, Fig. 6F) nor of interaction (F_2,41_ = 0.8, *p* = 0.47, Fig. 6F) was significant. PMA/ionomycin increased IFNγ expression across experimental groups (*p* < 0.001 for all, Fig. 6F). Similarly, TNFα expression was significantly affected only by PMA/ionomycin stimulation (F_1,41_ = 81.8, *p* < 0.001, Fig. 6G) with no significant effect of SPS/SPS + ARI treatment (F_2,41_ = 0.4, *p* = 0.69, Fig. 6G) or interaction (F_2,41_ = 0.5, *p* = 0.61, Fig. 6G). P*ost hoc* testing confirmed elevated TNFα expression in PMA/ionomycin stimulated splenocytes from all experimental groups (*p* < 0.001 for all, Fig. 6G). Analysis of interleukin 12 receptor beta (IL-12Rβ) expression revealed a significant effect of PMA/ionomycin stimulation (F_1,41_ = 16.5, *p* < 0.001, Fig. 6H), no effect of SPS/SPS + ARI treatment (F_2,41_ = 0.9, *p* = 0.43, Fig. 6H) but significant stimulation × treatment interaction (F_2,41_ = 5.2, *p* = 0.01, Fig. 6H). PMA/ionomycin selectively elevated IL-12Rβ mRNA levels in the SPS group (*p* < 0.001, Fig. 6H). Following PMA/ionomycin stimulation, SPS-derived splenocytes exhibited higher IL-12Rβ expression than those from VEH (*p* = 0.007, Fig. 6H). Importantly, ARI prevented this SPS-induced increase in IL-12Rβ expression (*p* = 0.017, Fig. 6H). For IL-17 A, two-ANOVA demonstrated a significant effect of PMA/ionomycin stimulation (F_1,43_ = 25.3, *p* < 0.001, Fig. 6I), whereas neither effect of SPS/SPS + ARI treatment (F_2,43_ = 0.6, *p* = 0.56, Fig. 6I) nor interaction (F_2,43_ = 1.2, *p* = 0.31, Fig. 6I) was significant. *Post hoc* analysis revealed increased IL-17 A expression following stimulation only in VEH (*p* = 0.016, Fig. 6I) and SPS (*p* < 0.001, Fig. 6I) groups. Signal transducer and activator of transcription 3 (STAT3) showed a similar profile, with a significant effect of PMA/ionomycin stimulation (F_1,39_ = 98.4, *p* < 0.001, Fig. 6J) but no significant effect of SPS/SPS + ARI treatment (F_2,39_ = 1.3, *p* = 0.29, Fig. 6J) or interaction (F_2,39_ = 2.1, *p* = 0.13, Fig. 6J). As shown by *post hoc* analysis, PMA/ionomycin significantly upregulated STAT3 expression in all experimental groups (*p* < 0.001 for all, Fig. 6J). Interestingly, unstimulated splenocytes from SPS + ARI rats exhibited higher STAT3 mRNA levels than those from SPS rats (*p* = 0.024, Fig. 6J). For the T helper 2 (Th2)-associated cytokine IL-4 (IL-4), two-way ANOVA showed a significant effect of PMA/ionomycin stimulation (F_1,31_ = 102.6, *p* < 0.001, Fig. 6K) while no effect of SPS/SPS + ARI treatment (F_2,31_ = 2.9, *p* = 0.070, Fig. 6K) or interaction (F_2,31_ = 0.1, *p* = 0.98, Fig. 6K). PMA/ionomycin robustly increased IL-4 expression in splenocytes from VEH, SPS, and SPS + ARI groups (*p* < 0.001 for all, Fig. 6K). To assess neutrophil activation, we measured myeloperoxidase (MPO) expression. Two-way ANOVA revealed no significant effects of PMA/ionomycin stimulation (F_1,36_ = 0.8, *p* = 0.39, Fig. 6L), SPS/SPS + ARI treatment (F_2,36_ = 0.5, *p* = 0.61, Fig. 6L), or their interaction (F_2,36_ = 1.4, *p* = 0.26, Fig. 6L).

### Correlations of neuroendocrine, immune, microbiota, and behavioral data

To evaluate potential interactions among splenic microbiota, neuroendocrine, behavioral, and immune parameters, we performed correlation analyses across all measured variables. We analyzed correlations using pooled data from all experimental groups (VEH, SPS, SPS + ARI) for the spleen, as well as for splenocytes stimulated ex vivo with LPS or PMA/ionomycin (including only the stimulated samples from VEH, SPS, and SPS + ARI).

**Fig. 7:**
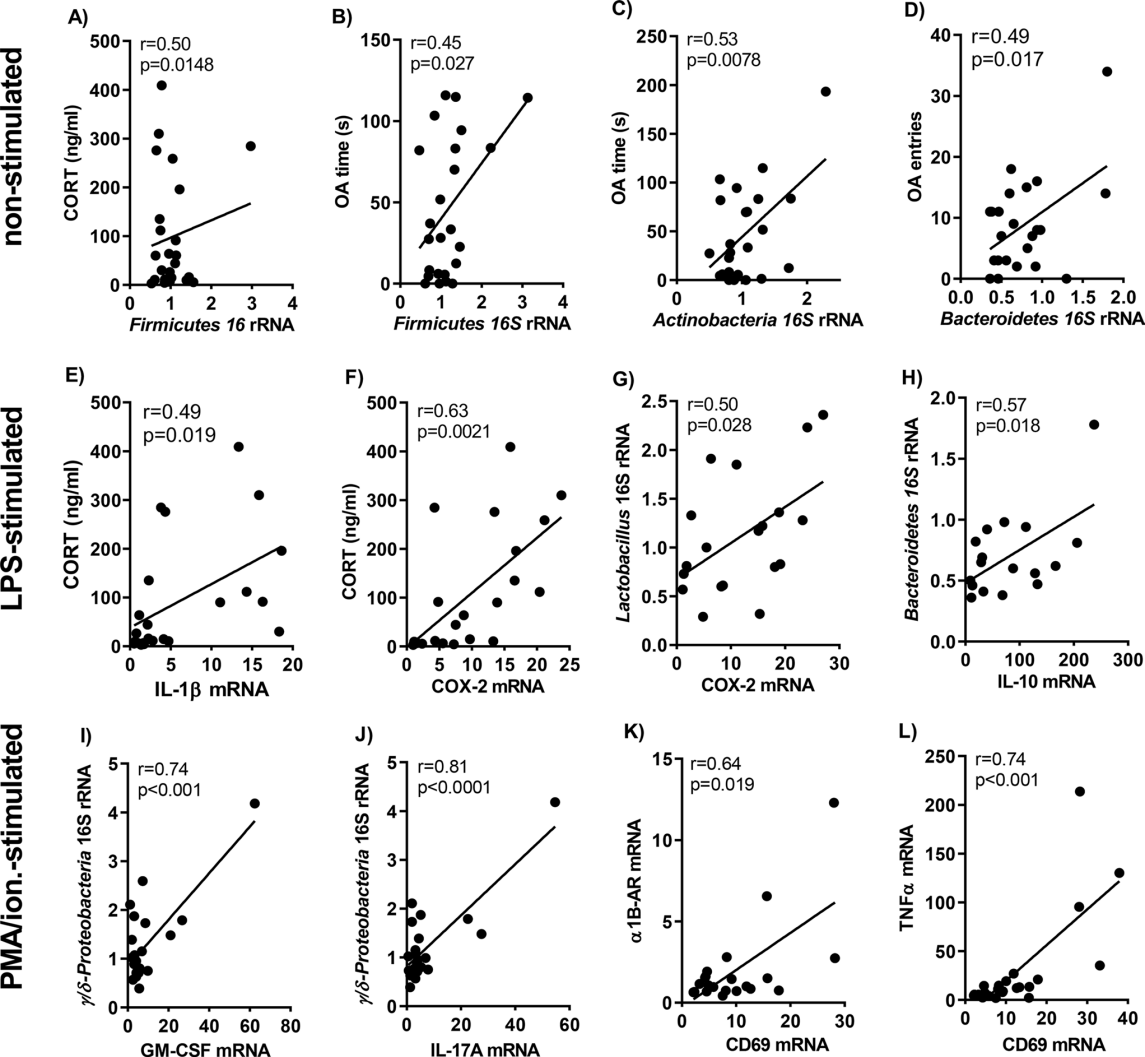
Correlations between spleen bacterial 16S rRNA levels, plasma corticosterone (CORT), and behavioral parameters (time spent in open arms (OA) of the elevated plus maze and number of OA entries; **A**–**D**). Correlations between CORT or spleen bacterial 16S rRNA levels and inflammatory mediators in splenocytes stimulated ex vivo with lipopolysaccharide (LPS, 10 µg/ml, E-H) or phorbol 12-myristate 13-acetate (PMA, 81 nmol/l) and ionomycin (1.34 µmol/l) (I–L) for 3 h. Analyses were performed using pooled data from all experimental groups (VEH, SPS, SPS + ARI). Unstressed rats were treated with vehicle (VEH; 2% Tween 20 in saline; *n* = 8–10) for 28 days. Rats exposed to SPS received vehicle (SPS; *n* = 8–10) or ARI (5 mg/kg) (SPS + ARI; *n* = 6–7) for 28 days. CORT levels were determined by ELISA. Gene expressions and 16s rRNA levels were quantified by qRT-PCR, normalized to RPS29, and expressed as fold change vs. control. Pearson’s correlation coefficients (r) and p-values are shown. Abbreviations: 16S rRNA, 16 S subunit of ribosomal ribonucleic acid; α1B-AR, alpha 1B adrenergic receptor; ARI, aripiprazole; CD69, cluster of differentiation 69; CORT, corticosterone; COX-2, cyclooxygenase-2; ELISA, Enzyme-linked immunosorbent assay; GM-CSF, granulocyte-macrophage colony-stimulating factor; IL-1β, interleukin 1 beta; IL-10, interleukin 10; IL-17 A, interleukin 17 A; LPS, lipopolysaccharide; OA, open arms; PMA, phorbol 12-myristate 13-acetate; SEM, standard error of the mean; SPS, single prolonged stress; TNFα, tumor necrosis factor alpha; VEH, vehicle

We found positive correlation of *Firmicutes* rRNA and plasma CORT (*p* = 0.0148, *r* = 0.5, Fig. 7A). Behavioral data, namely time that rats spent in the open arms of the EPM correlated positively with *Firmicutes* rRNA (*p* = 0.027, *r* = 0.45, Fig. 7B) and *Actinobacteria* rRNA (*p* = 0.0078, *r* = 0.53, Fig. 7C) and entries to open arms of the EPM with phylum *Bacteroidetes* rRNA with (*p* = 0.017, *r* = 0.49, Fig. 7D). Plasma CORT level positively correlated with expression of IL-1β (*p* = 0.014, *r* = 0.58, Fig. 7E) and COX-2 (*p* = 0.0021, *r* = 0.63, Fig. 7F) mRNA in LPS-stimulated splenocytes. From bacteria, *Lactobacillus* rRNA correlated with COX-2 (*p* = 0.028, *r* = 0.5, Fig. 7G), while *Bacteroidetes* rRNA correlated with IL-10 mRNA (*p* = 0.018, *r* = 0.57, Fig. 7H) in LPS-stimulated splenocytes. *γ/δ-Proteobacteria* rRNA correlated with PMA/ionomycin-stimulated levels of GM-CSF (*p* < 0.001, *r* = 0.74, Fig. 7I) and IL-17 A mRNA (*p* = 0.018, *r* = 0.57, Fig. 7J). Expression of activation marker CD69 correlated with expression of α1B adrenoreceptors (*p* = 0.019, *r* = 0.64, Fig. 7K) and with TNFα mRNA (*p* < 0.001, *r* = 0.74, Fig. 7L).

## Discussion

In the present study, we showed that SPS induces coordinated changes in spleen microbiota, neuroendocrine activity, and immune responses associated with stress-related behavior, while ARI partially mitigates these effects, highlighting spleen bacterial translocation as a potential contributor to PTSD-like pathology.

Although stress and antipsychotic treatment are known to disrupt the delicate balance between beneficial and pathogenic gut bacteria [34, 35], bacterial translocation and the spleen microbiota profile remain, to our knowledge, uncharacterized in the SPS model and following ARI treatment. We detected bacterial 16 S rRNAs in the spleen, including predominant gut microbiota phyla *(Firmicutes*, *Bacteroidetes*, *Actinobacteria*, and *Proteobacteria*) and genera known to influence immune responses [36]. While numerous studies have documented stress-induced changes in gut microbiota composition based on fecal samples, only a few have investigated bacterial translocation into the bloodstream and peripheral organs such as the liver or spleen that may not only reflect the gut microbiota composition but also indicate the degree of gut permeability [36].

Although in SPS-exposed rats we did not find altered total bacterial load or the abundance of the major phylum *Firmicutes* in the spleen, they exhibited decreased levels of 16 S rRNA of the second most abundant phylum, *Bacteroidetes*.

It is well established that stress can increase the *Firmicutes/Bacteroidetes* ratio [37] while decreasing the proportion of *Bacteroidetes* relative to *Proteobacteria* in the gut microbiota [38, 39]. Conversely, exposure to SPS resulted in a significant upregulation of *Lactobacillus* and γ/δ-*Proteobacteria* 16 S rRNA levels in the spleen, whereas administration of ARI completely abolished this response. The effects of ARI on the gut microbiota have been previously characterized, showing that ARI significantly decreases the relative abundances of the phyla *Firmicutes* and *Actinobacteria*, while increasing *Proteobacteria* [19]. Although we could not monitor the effect of ARI on microbiota itself, but just in the SPS context, we could see that ARI was able to suppress some of the SPS-elicited alterations (*Proteobacteria* and *Lactobacillus)*.

We further established associations between the spleen microbiota, neuroendocrine stress response, and splenic immune function. We found a positive correlation between *Firmicutes* 16 S rRNA levels and plasma CORT level what indicates a potential link between spleen-resident bacteria and the systemic stress response. Increased bacterial translocation into the spleen might indicate stronger HPA axis response and greater susceptibility to stress, as interaction between microbiota and neuroendocrine stress response has been intensively studied primarily from the perspective of stress susceptibility or resilience [40].

Moreover, we found correlations between 16 S rRNA levels of specific bacterial taxa and manifestations of anxiety-like behavior, suggesting an association between the anxiety-like phenotype and bacterial translocation. The time rats spent in the OA and the number of OA entries positively correlated with spleen 16 S rRNA levels specific for *Firmicutes*, *Actinobacteria*, and *Bacteroidetes.* These taxonomic groups predominantly include beneficial bacteria that are frequently diminished in the gut following stress exposure, but in the spleens of PTSD-like rats, we observed only decreased levels of *Bacteroidetes* 16 S rRNA.

PTSD symptoms are often associated with systemic inflammation and elevated levels of inflammatory mediators. While PTSD models using social stressors are well characterized, the inflammatory mechanisms underlying behavioral outcomes in models, such as predator stress or SPS, remain poorly understood [41].

The SPS model we used does not fully replicate features of social stressors such as repeated social defeat stress (RSDS), as we did not observe upregulation in the expression of common inflammatory cytokines or markers associated with immune cell recruitment in the spleen. Therefore, we stimulated splenocytes ex vivo with LPS and PMA/ionomycin to induce cytokine production, enabling evaluation of their immune and inflammatory responses as well as characterization of specific cellular phenotypes.

LPS stimulation did not elicit an enhanced response of splenocytes in SPS-exposed rats, suggesting that splenic myeloid populations such as monocytes, macrophages, dendritic cells, and neutrophils may play a limited role in the SPS model. This is in contrast with findings from chronic/repeated stress models, such as repeated social defeat stress, where myeloid cell recruitment is commonly observed [4, 41, 42], or with our previous findings, in which 7-day repeated immobilization stress potentiated the splenic inflammatory response to acute LPS stimulation [43]. Although SPS did not affect the overall response to LPS, a positive correlation was observed between LPS-induced expression of IL-1β and COX-2 with plasma corticosterone, indicating a possible influence of neuroendocrine activity on the modulation of splenocyte inflammatory responses in the SPS model. We also observed a positive correlation of anti-inflammatory cytokine IL-10 in LPS-stimulated splenocytes with spleen *Bacteroidetes* 16 S rRNAs, which is in accordance with previously described assumed anti-inflammatory effects of gut *Bacteroidetes* [44, 45]. This finding also aligns with the generally observed reduction of *Bacteroidetes* abundance in the gut following stress, which may contribute to a diminished anti-inflammatory response [44]. The positive correlation between spleen *Lactobacillus* 16 S rRNA levels and COX-2 expression in LPS-stimulated splenocytes closely aligns with the findings that translocation of commensal *Lactobacillus* species to the spleen might prime the innate immune system for enhanced reactivity [36].

Ex vivo stimulation with PMA/ionomycin revealed an effect of SPS on cell activation (CD69 expression) or cytokine responses characteristic of various T-helper cell phenotypes, whereas ARI treatment attenuated some of the changes induced by SPS. Specifically, SPS appeared to potentiate Th1- and Th17-associated markers while slightly suppressing Th2-associated IL-4 expression, suggesting an overall shift toward a pro-inflammatory phenotype.

Several animal studies have demonstrated augmented Th1 and Th17 immune response in PTSD models, highlighting the involvement of these T-helper cell subsets in stress-induced neuroinflammation and behavioral alterations [7, 46]. Findings from an RSDS model suggest that increased sympathetic activity within the spleen may contribute to the pro-inflammatory, Th17-mediated immune response characteristic of PTSD pathology [7]. In this context, T cell–intrinsic TH–dependent catecholamine production may represent a critical regulator of the stress-induced Th17 response driving the pro-inflammatory phenotype. Furthermore, a recent study by Lauten et al. [31] demonstrated that adrenergic signaling through β-adrenergic receptors and cAMP pathways modulates Th17/Treg differentiation, further supporting the link between catecholaminergic activity and immune dysregulation in stress-related disorders. Given that ARI treatment attenuated SPS-induced IL-17 A expression, it is possible that dopamine synthesized by TH-expressing T cells participates in this process and that ARI exerts its effect by dampening excessive dopaminergic signaling. As we observed slightly reduced expression of several Th1- and Th17-related genes in the SPS group treated with ARI, it is also reasonable to consider the potential immunosuppressive effects of ARI on Th1 and Th17 lymphocyte subtypes, as previously described [47, 48]. Notably, the expression of Th17-related cytokines, i.e., IL-17 A and GM-CSF, in splenocytes ex vivo stimulated with PMA/ionomycin correlated with 16 S rRNA sequences corresponding to the γ/δ-classes of *Proteobacteria*. Given the correlations between *Proteobacteria* rRNA and molecules predominantly produced by activated Th17 cells, we assume a possible role for these bacteria in mediating Th17-driven symptoms associated with PTSD.

On the other hand, in the SPS rats, the PMA/ionomycin stimulation induced expression of the inflammatory mediators COX-2 and IL-1β in VEH-treated rats, but this was effectively abolished by ARI treatment. Considering that neutrophils, as primary producers of IL-1β among PMA/ionomycin-activated cells, are critically involved, we assume that the anti-inflammatory effect of ARI may be mediated through its selective cytotoxic action on neutrophils, which would be consistent with previous clinical case reports that showed ARI-induced neutropenia and leukocytopenia [49–51]. MPO expression, a neutrophil-specific marker, exhibited a comparable pattern of modulation, further substantiating this hypothesis.

Altogether, our findings showed that the SPS elicits immunomodulatory alterations in the spleen that closely interact with the changes in the neuroendocrine system and spleen microbiota.

## Conclusions

In conclusion, this study highlights the spleen as an important integrative organ in the interactions among the microbiota, the immune system, and the neuroendocrine stress response in a PTSD model. SPS induced specific alterations in spleen-associated bacterial taxa that were closely linked to corticosterone level, anxiety-like behavior, and immune activity. These findings suggest that bacterial translocation to the spleen is functionally relevant for stress-related outcomes. Moreover, SPS promoted a pro-inflammatory Th1/Th17 profile, partially attenuated by ARI. Overall, our results underscore the important role of the spleen in mediating microbiota–immune–neuroendocrine crosstalk in the SPS model of PTSD.

### Limitations and future direction

This study has several limitations that should be addressed in future research. First, we analyzed only selected bacterial taxa in the spleen, providing limited insight into the full splenic microbiota, which could be better characterized using comprehensive bacterial DNA sequencing. Importantly, our data do not allow us to determine the origin of the observed changes in splenic microbiota. Specifically, we cannot distinguish whether these changes result from stress-induced alterations in gut microbiota composition, increased bacterial translocation due to changes in intestinal permeability, or altered bacterial clearance at the intestinal barrier or within the spleen. A more detailed assessment of gut microbiota across different intestinal regions, intestinal barrier integrity, and bacterial translocation markers would help clarify these mechanisms.

Immune cell analyses using higher-resolution methods, such as flow cytometry, could more precisely define splenocyte population shifts. While cytokine and immune factor expression aligned with previous reports, protein-level confirmation is needed. Moreover, we did not study the effect of aripiprazole in unstressed rats as the study was primarily aimed to study the ARI effect on the changes induced by SPS, not how ARI itself modifies measured parameters in healthy rats. Additionally, the study was limited to male rats, precluding the assessment of sex-specific effects. Although sex differences in behavioral and physiological responses to trauma have been reported in the SPS model, their investigation was beyond the scope of the present study, which was designed to minimize variability and maximize the detection of treatment-related effects.

## Data Availability

The data that support the findings of this study are available from the corresponding author upon reasonable request.

## Abbreviations

16S rRNA: 16 S subunit of ribosomal ribonucleic acid
α1B-AR: Alpha 1B adrenergic receptor
α2A-AR: Alpha 2 A adrenergic receptor
β1-AR: Beta 1 adrenergic receptor
β2-AR: Beta 2 adrenergic receptor
ARI: Aripiprazole
CCL2: C-C motif chemokine ligand 2
CCL21: C-C motif chemokine ligand 21
CD4^+^: Cluster of differentiation 4^+^
CD40: Cluster of differentiation 40
CD69: Cluster of differentiation 69
CD80: Cluster of differentiation 80
CHAT: Choline acetyltransferase
COX-2: Cyclooxygenase-2
CORT: Corticosterone
ELISA: Enzyme-linked immunosorbent assay
EPM: Elevated plus maze
GM-CSF: Granulocyte-macrophage colony-stimulating factor
GR: Glucocorticoid receptor
HPA axis: Hypothalamic–pituitary–adrenal axis
IFNγ: Interferon gamma
IL-1β: Interleukin 1 beta
IL-2: Interleukin 2
IL-4: Interleukin 4
IL-6: Interleukin 6
IL-10: Interleukin 10
IL-12A: Interleukin 12 A
IL-12Rβ: Interleukin 12 receptor beta
IL-17A: Interleukin 17 A
ip: Intraperitoneally
LPS: Lipopolysaccharide
LY6C: Lymphocyte antigen 6 complex
MPO: Myeloperoxidase
OA: Open arms of the elevated plus maze
PMA: Phorbol 12-myristate 13-acetate
PTSD: Post-traumatic stress disorder
qRT-PCR: Quantitative real-time polymerase chain reaction
RPS29: Ribosomal protein S29
RSDS: Repeated social defeat stress
SIGNR1: Specific intercellular adhesion molecule-3-grabbing non-integrin receptor 1
SPS: Single prolonged stress
STAT3: Signal transducer and activator of transcription 3
TH: Tyrosine hydroxylase
Th1: T helper 1 cells
Th2: T helper 2 cells
Th17: T helper 17 cells
TNFα: Tumor necrosis factor alpha
VEH: Vehicle
